# Case Report: Successful delivery following chemotherapy in a pregnant patient with metastatic SMARCB1-deficient renal medullary carcinoma

**DOI:** 10.3389/fonc.2025.1606647

**Published:** 2025-08-29

**Authors:** Panayiotis D. Kontoyiannis, Adam Kuykendal, Chad Tang, Jessica P. Cheng, Beei Chan, Susan S. Thomas, Priya Rao, Bora Lim, Pavlos Msaouel

**Affiliations:** ^1^ McGovern Medical School, UT Houston Health Science Center, Houston, TX, United States; ^2^ Department of Medical Oncology, Novant Health Cancer Institute – Ballantyne, Charlotte, NC, United States; ^3^ Division of Radiation Oncology, Department of Radiation Oncology, The University of Texas MD Anderson Cancer Center, Houston, TX, United States; ^4^ Department of Translational Molecular Pathology, Pathology and Laboratory Medicine, The University of Texas MD Anderson Cancer Center, Houston, TX, United States; ^5^ Division of Cancer Medicine, Department of Genitourinary Medical Oncology, The University of Texas MD Anderson Cancer Center, Houston, TX, United States; ^6^ Division of Pathology and Laboratory Medicine, Department of Pathology, The University of Texas MD Anderson Cancer Center, Houston, TX, United States; ^7^ Division of Cancer Medicine, Department of Breast Medical Oncology, The University of Texas MD Anderson Cancer Center, Houston, TX, United States; ^8^ UTHealth Houston Graduate School of Biomedical Sciences (GSBS), Houston, TX, United States; ^9^ David H. Koch Center for Applied Research of Genitourinary Cancers, The University of Texas MD Anderson Cancer Center, Houston, TX, United States

**Keywords:** renal cell carcinoma unclassified with medullary phenotype, pregnancy, SMARCB1 loss, chemotherapy, case report

## Abstract

SMARCB1-deficient renal medullary carcinoma (RMC) is a rare and aggressive kidney cancer defined by the loss of SMARCB1 tumor suppressor and primarily affecting adolescents and young adults with sickle hemoglobinopathies. Approximately 7% of RMC cases, known as renal cell carcinoma unclassified with medullary phenotype (RCCU-MP), lack sickle hemoglobinopathy. RMC does not respond to immune checkpoint inhibitors and antiangiogenic tyrosine kinase inhibitors, with chemotherapy being the main treatment. Here we present the first documented case of RMC diagnosed during pregnancy. A 24-year-old woman presented with right-sided back pain, leading to the discovery of a 6-cm right renal mass. Pathology confirmed RCCU-MP with SMARCB1 loss. With the woman at 16 weeks into pregnancy, imaging revealed metastatic retroperitoneal lymphadenopathy and lung nodules. A chemotherapy regimen of doxorubicin and cyclophosphamide, followed by weekly paclitaxel, was selected for safety in pregnancy. This approach yielded significant tumor shrinkage and alleviated the symptoms, allowing for the safe, preterm delivery of a healthy baby at 33 weeks. Following delivery, the patient received combination chemotherapy and definitive radiation therapy, achieving disease control. At 2 years post-diagnosis, she remains alive, exceeding the median survival for RCCU-MP. This case demonstrates that established chemotherapeutic regimens used in pregnant patients with other cancers can be successfully applied to manage RMC during pregnancy. Our findings underscore the importance of early, aggressive treatment and suggest that a coordinated approach can achieve favorable outcomes for both the mother and the fetus.

## 1 Introduction

SMARCB1-deficient renal medullary carcinoma (RMC) is a rare but aggressive subtype of renal cell carcinoma (RCC) that primarily affects adolescents and young adults (AYAs) with underlying sickle hemoglobinopathies, such as sickle cell trait and sickle cell disease (1–4). However, approximately 7% of RMC cases are not associated with hemoglobinopathies and are classified as renal cell carcinoma unclassified with medullary phenotype (RCCU-MP) (1, 3, 5). All RMC cases, including RCCU-MP, are defined by the loss of SMARCB1 tumor suppressor protein expression as determined by immunohistochemistry (IHC) (1, 6). In contrast to most other RCCs, RMC is resistant to currently approved immune checkpoint therapies (7) and tyrosine kinase inhibitors (TKIs) targeting the vascular endothelial growth factor (VEGF) pathway (4, 8). Platinum-based cytotoxic chemotherapy is the recommended first-line therapy for RMC (1). Combination chemotherapy with definitive radiation may also be used in selected patients with oligoprogressive or oligometastatic RMC (9). Herein we report the first documented case of RMC during pregnancy in a young woman who was diagnosed with cancer on the same day she discovered she was pregnant. The tailored management and treatment plan enabled a healthy preterm delivery that can guide the clinical approach for future cases of pregnant patients with metastatic RMC.

## 2 Case presentation

A 24-year-old Caucasian female with no major past medical or surgical history presented to primary care clinic with a 1-week history of right-sided lower back and flank pain, scored 4/10 in severity. She denied fevers, chills, or gross hematuria. In the clinic, a point-of-care urine dipstick showed trace blood and a computed tomography (CT) of the abdomen and pelvis without contrast was performed to rule out kidney stones. The patient was instructed to use ibuprofen and acetaminophen for pain.

The CT of the abdomen and pelvis showed two punctuate stones in the left kidney and, despite being limited by lack of contrast, showed findings suggestive of pyelonephritis and/or a renal abscess. The urine cultures were negative, but the patient was treated empirically with ciprofloxacin due to concern for infection. She was then referred to a urologist, and these findings were attributed at that visit as papillary necrosis secondary to ibuprofen use. A renal ultrasound (US) was ordered for repeat imaging in 3 months.

At 2 weeks later, the flank pain had become bilateral, and the patient began reporting gross hematuria. She underwent renal US which showed a 5.9-cm right renal mass. Subsequent magnetic resonance imaging (MRI) with and without contrast confirmed the presence of a 6-cm mass suspicious for RCC. At 2 weeks later, the patient underwent an enhanced recovery after surgery (ERAS) hand-assisted laparoscopic nephrectomy of her right kidney with no complications. In the morning of her surgery, the patient had a urine pregnancy test that came back positive. The last menstrual period (LMP) was exactly 4 weeks prior. A subsequent US evaluation confirmed this to be an intrauterine pregnancy.

**Figure 1:**
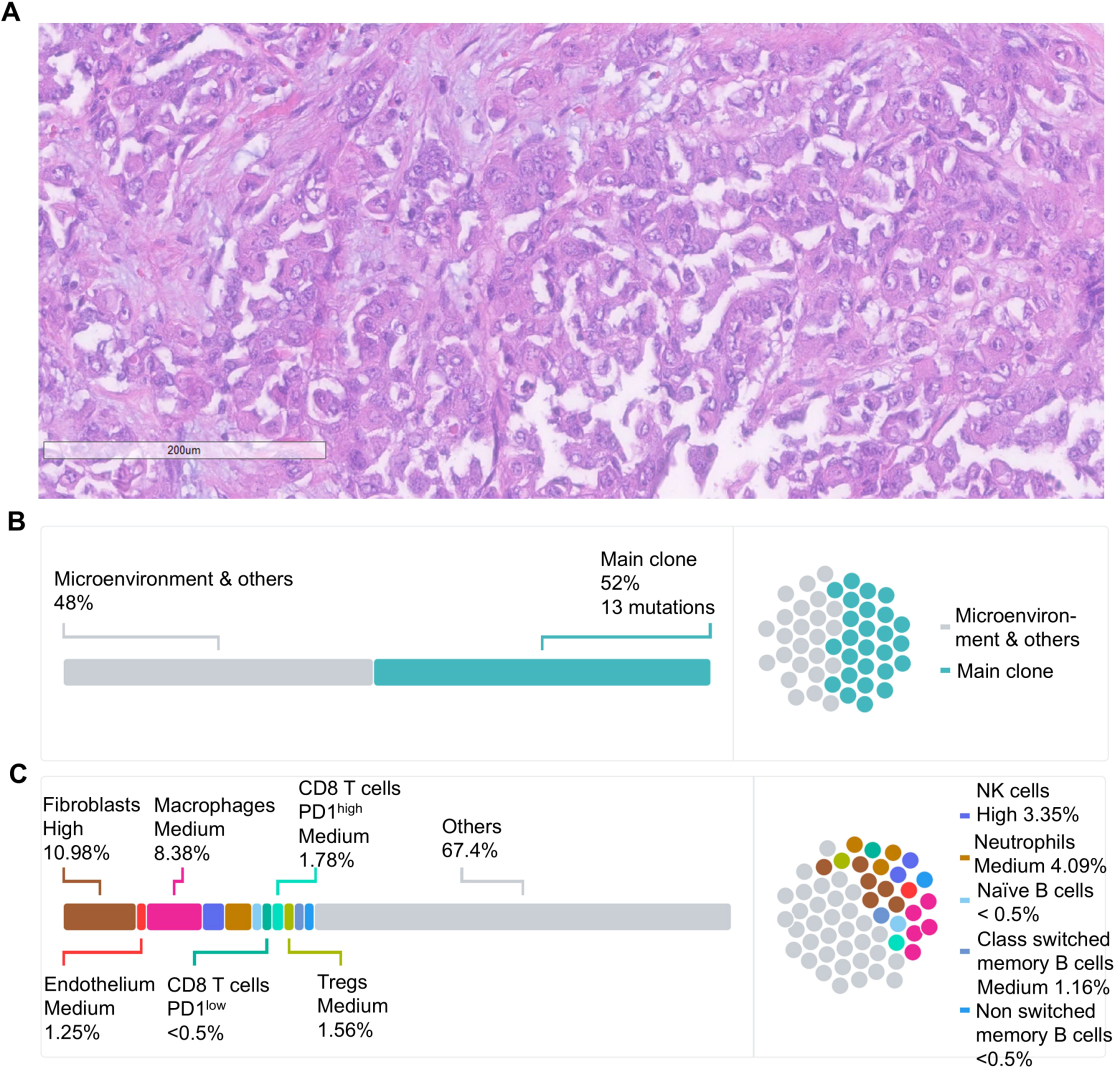
**(A)** Histological evaluation by hematoxylin and eosin staining showed a high-grade carcinoma without sarcomatoid or rhabdoid features. The malignant cells were epithelioid, featuring large nuclei with vesicular chromatin, nucleoli that ranged from inconspicuous to prominent, and abundant amphophilic cytoplasm consistent with RMC. Scale bar = 200 μm. **(B)** Schematic representation of the tumor clonal composition based on whole-exome sequencing of the nephrectomy tumor specimen and germline control. Major tumor clones are presented as a percentage from the entire tumor tissue. **(C)** Schematic representation of the cellular makeup of the tumor micro-environment (TME) based on the RNAseq of the nephrectomy specimen. The composition of malignant and microenvironment compartments is inferred from gene expression profiling as previously described (10).

The pathology evaluation of the nephrectomy specimen showed a high-grade carcinoma without sarcomatoid or rhabdoid features, 5.9 cm in greatest dimension and invading the renal sinus and perinephric tissue (pT3a). There was no lymphovascular invasion, and the resection margins were free of tumor. The lymph nodes were not sampled. Morphologically, the malignant cells were epithelioid with large nuclei with vesicular chromatin and variably conspicuous nucleoli and abundant amphophilic cytoplasm. Numerous mitotic figures and apoptotic debris were present (**Figure 1A**). The IHC showed the malignant cells to be positive for PAX8, CK7, and CK20 (weak) while negative for CD10, p63, GATA-3, and SMARCB1 (INI-1). The hemoglobin electrophoresis came back negative for the sickle cell trait or any other hemoglobinopathy, confirming the diagnosis as RCCU-MP.

Whole-exome sequencing (WES) and bulk RNA-sequencing (RNA-seq) were performed on formalin-fixed paraffin-embedded (FFPE) tissue from the nephrectomy specimen as previously described (11, 12). The microsatellite status was stable, and the tumor mutational burden was low at 0.43 mut/Mb, as is typically observed in RMC (6). Somatic missense mutations were identified in 13 genes (**Table 1**). As is often the case in RMC (6), no SMARCB1 mutations were detected, but there was a homozygous copy number loss of the *SMARCB1* gene. The tumor cells comprised a single dominant clone with no detectable subclones (**Figure 1B**). The RNA-seq revealed a high expression of genes known to be upregulated in RMC such as CD70, EGFR, and MUC16 (6) as well as a moderate expression of PD-L1. Cell content deconvolution using the Kassandra algorithm (10) revealed enrichment for cancer-associated fibroblasts and macrophages in the tumor microenvironment (TME), with few CD8 T cells expressing high PD-1 levels (**Figure 1C**).

**Table 1:** List of somatic variants identified according to validated thresholds for FFPE samples based on whole-exome sequencing of the nephrectomy tumor specimen and germline control.

Gene	DNA mutation	Amino acid alteration	Mutation type	Variant allele frequency	Clonality status
PTPRC	c.3598G>C	p.Val1200Leu	Missense	27.3%	Clonal
SRBD1	c.1609C>T	p.Arg537Cys	Missense	25.6%	Clonal
TTN	c.52142T>C	p.Ile17381Thr	Missense	29%	Clonal
FBXL17	c.1757T>C	p.Val586Ala	Missense	18.9%	Clonal
DACT2	c.343G>A	p.Gly115Arg	Missense	23.1%	Clonal
NOM1	c.674C>G	p.Ser225Cys	Missense	29.9%	Clonal
CHAT	c.1345G>A	p.Val449Ile	Missense	29.8%	Clonal
PAH	c.687C>G	p.Asp229Glu	Missense	21.6%	Clonal
SUPT20H	c.905T>G	p.Ile302Arg	Missense	13.4%	Clonal
MIS18BP1	c.3265A>G	p.Thr1089Ala	Missense	21.5%	Clonal
BICDL2	c.475C>T	p.Arg159Trp	Missense	66.7%	Clonal
NLRP11	c.94C>T	p.Arg32Cys	Missense	23.9%	Clonal
DYRK1A	c.1286G>A	p.Arg429His	Missense	28.2%	Clonal

The mutation, mutation type, and variant allele frequency are listed for each variant.

**Figure 2:**
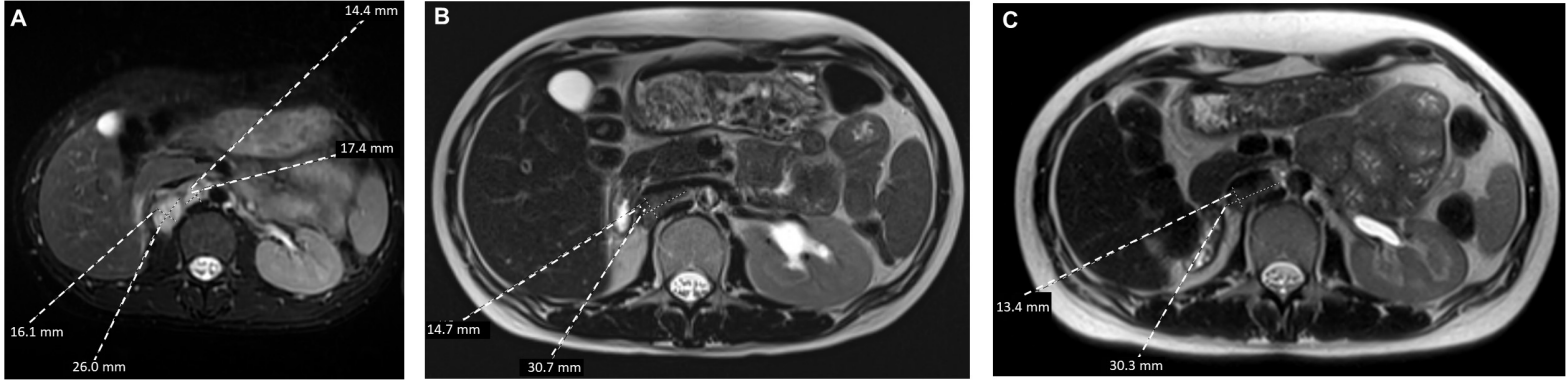
Axial T2-weighted MRI view of the retroperitoneal metastasis **(A)** at baseline, **(B)** following four cycles of doxorubicin plus cyclophosphamide, and **(C)** following six infusions of weekly paclitaxel.

**Figure 3:**
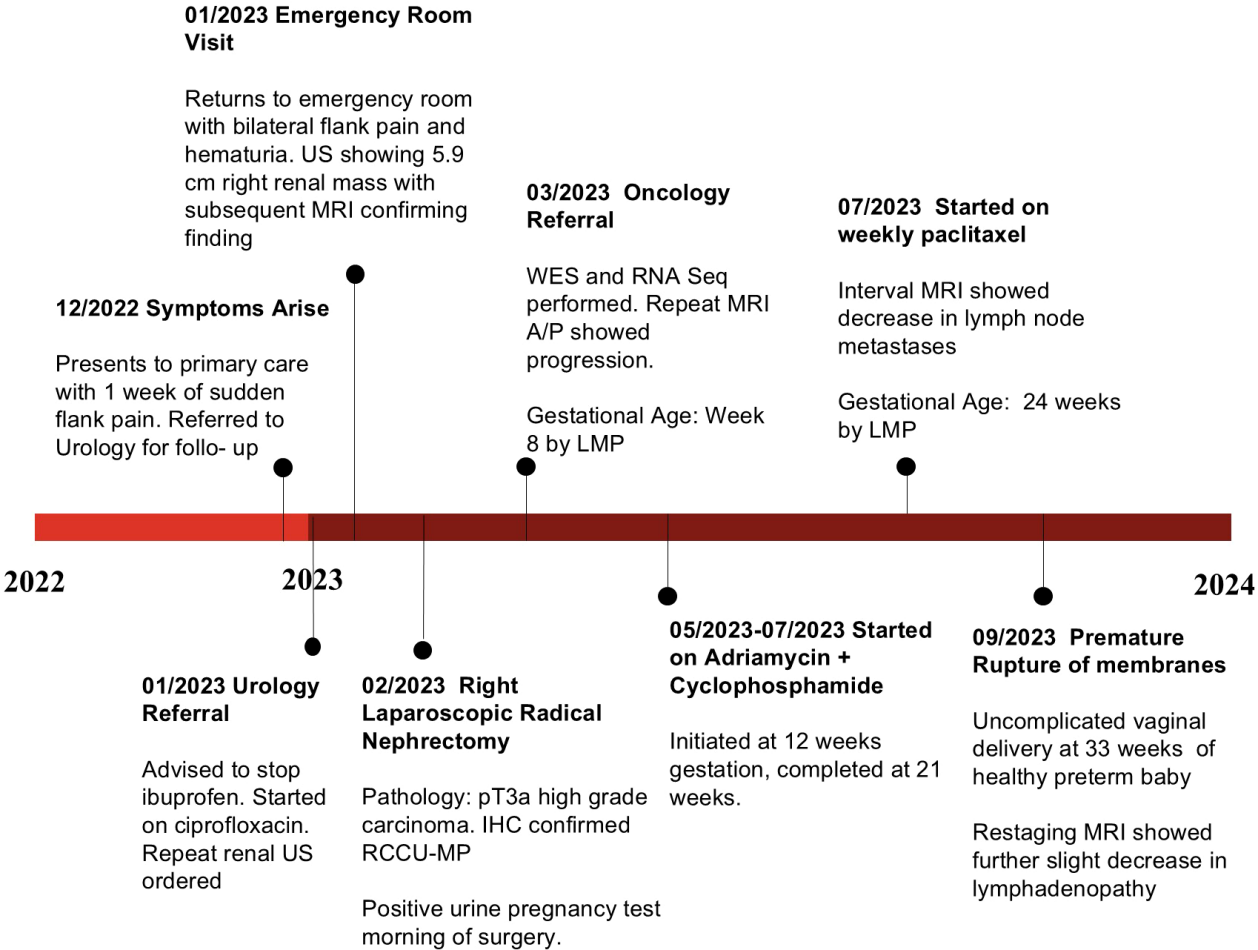
Timeline of the patient’s clinical course from initial symptom onset to delivery, highlighting key diagnostic and therapeutic milestones.

At approximately 6 weeks after the nephrectomy, a repeat MRI of the abdomen and pelvis without contrast revealed enlarged retrocaval and aortocaval lymph nodes up to 4.2 cm in maximal diameter (**Figure 2A**) consistent with metastatic retroperitoneal lymphadenopathy, the most common site of metastasis in RMC (3). The non-contrast CT of the chest showed enlarging lung nodules of up to 6 mm in diameter suspicious for metastatic disease. No evidence of central nervous system metastasis was noted on MRI of the brain. The patient was in her 16th week of pregnancy. Clinically, she began experiencing worsening right-sided abdominal pain. The decision was made to start chemotherapy consisting of the AC-T regimen (four cycles of doxorubicin plus cyclophosphamide followed by weekly paclitaxel), which has established safety profile in pregnant patients with breast cancer (13, 14). RMC is sensitive to doxorubicin (6, 15) and paclitaxel (6, 16). Although cyclophosphamide is not typically used to treat RMC (3, 16), the loss of SMARCB1 is known to increase replication stress, rendering tumor cells susceptible to alkylating agents such as cyclophosphamide (6, 17). The chemotherapy regimen initially consisted of doxorubicin at 60 mg/m^2^ given intravenously (IV) in combination with cyclophosphamide at 600 mg/m^2^ IV every 3 weeks for four cycles. The premedication antiemetics consist of ondansetron at 8 mg IV and famotidine at 20 mg IV. If necessary, fosaprepitant at 150 mg IV and metoclopramide could be added as needed. The baseline transthoracic echocardiogram showed normal left ventricular size and systolic function with left ventricular ejection fraction calculated as 57% using the bi-plane method of disks. The patient’s abdominal pain subsided after two cycles of this chemotherapy regimen and she did not experience any adverse events, while the anatomical scans of the fetus at 20 weeks of gestation showed normal development. The fourth cycle of doxorubicin plus cyclophosphamide was completed at week 25 of pregnancy with re-staging of MRI of the abdomen and pelvis without contrast, showing a significant interval decrease in the size of the retrocaval/retroperitoneal lymphadenopathy (**Figure 2B**). For example, the confluent retrocaval lymphadenopathy decreased in size from 4.2 × 2.4 cm to 3.1 x× 1.5 cm. A non-contrast CT of the chest also showed a decrease in the size of the multiple bilateral lung nodules. At week 28 (3 weeks after completion of the last cycle of doxorubicin plus cyclophosphamide), weekly paclitaxel at 80 mg/m^2^ for up to six doses was initiated with the goal to not continue chemotherapy after 34 weeks of pregnancy. The patient received five doses of weekly paclitaxel with no issues. However, at week 33, she experienced a premature rupture of membranes and subsequently had an uncomplicated spontaneous vaginal delivery of a healthy premature baby. Restaging non-contrast MRI of the abdomen and pelvis and CT of the chest, a further slight decrease in metastatic disease is shown (**Figure 2C**). Following the successful pregnancy, the patient was started on definitive radiation therapy in combination with carboplatin plus paclitaxel for her persistent oligometastatic disease. The timeline of diagnosis and pregnancy are outlined in **Figure 3**. She remains alive and with good disease control 2 years after her diagnosis.

## 3 Discussion

Although the present case is the first description of successful delivery in a pregnant patient with metastatic RMC, it will not be the last as the median age of RMC diagnosis overlaps with peak fertility age (3). Although rare, RMC is still the third most commonly diagnosed RCC in the AYA population in the United States (18). Our case demonstrates that utilizing established therapies, which have been well tested in pregnant patients with other cancer types (13, 14), can enable patients to continue their intrauterine pregnancy without needing to consider termination. Had the patient not been pregnant, active cytotoxic chemotherapies such as platinum salts, paclitaxel, gemcitabine, or doxorubicin would be deployed (1, 3, 6, 17). While platinum-based chemotherapy is not absolutely contraindicated in pregnancy, it carries the risk of fetal bone marrow suppression and ototoxicity (19). Although carboplatin carries less ototoxicity risk than cisplatin, studies in primates have shown high carboplatin transplacental transfer of carboplatin to the fetus with mean fetal plasma concentrations reaching 57.5% of maternal concentrations (20). Paclitaxel is a therapeutic mainstay in the first-line therapy of RMC and has an excellent safety profile in pregnancy, in part because it is a substrate for p-glycoprotein which protects they fetus by carrying paclitaxel from the fetal to the maternal side of the placental barrier (19). As a result, transplacental transfer of paclitaxel to the fetus is only marginal, with primate studies showing that the mean fetal plasma concentrations are 1.5% of maternal concentrations (20). SMARCB1 loss induces replication stress, making RMC cells susceptible to topoisomerase II inhibitors such as doxorubicin (6, 15, 17) and potentially to alkylating agents such as cyclophosphamide that form DNA crosslinks, blocking the replication fork progression and leading to increased replication stress (21, 22). Nucleoside analogs such as gemcitabine may also target replication stress (6, 15, 17), and while there are case reports of their successful use in pregnancy (23, 24), we chose to avoid it due to its potential for teratogenicity and genotoxicity. Doxorubicin, cyclophosphamide, and paclitaxel are commonly used in pregnant patients with breast cancer, which is the most common solid malignancy treated with cytotoxic chemotherapy in AYA women of childbearing age (25) and therefore has the most extensive and long-term safety data available (13, 14, 26). Studies in primates have shown that mean fetal plasma concentrations compared to maternal concentrations is 7.5% for doxorubicin and 6.3% for cyclophosphamide (27). The ACT regimen we chose is commonly used and easily applied in the community center, thus alleviating the need for the patient to travel to our tertiary center during her pregnancy. Combination chemotherapy with definitive radiation therapy is a promising treatment modality for patients with oligoprogressive or oligometastatic RMC (9). However, such an approach should be deferred until after delivery due to the short- and long-term risks of fetal radiation exposure, particularly in scenarios where the metastatic disease is not located sufficiently far from the fetus (28). Our use of the AC-T chemotherapy regimen during pregnancy produced immediate clinical and radiological responses, allowing for safe delivery followed by the use of combination chemotherapy with definitive radiation. Notably, our patient has survived for longer than 2 years from diagnosis, exceeding the median RCCU-MP overall survival of 19.5 months (3). Given the aggressiveness of RMC, including the RCCU-MP subtype, it is unlikely that either she or her fetus would have survived without timely initiation of chemotherapy during pregnancy.

## 4 Conclusion

In conclusion, this case report provides a unique account of managing metastatic RMC during pregnancy, emphasizing the importance of a tailored, multidisciplinary approach. The use of well-established chemotherapy regimens with demonstrated safety profiles in pregnant patients, such as the AC-T regimen, allowed for disease control and a successful preterm delivery. This case highlights the potential of leveraging known therapeutic strategies from other malignancies to manage RMC in pregnant patients while minimizing risks to the fetus. The patient’s prolonged survival beyond the typical outcomes for RMC underscores the effectiveness of prompt, innovative management. As more cases emerge, our experience can inform future clinical decisions and improve the outcomes for pregnant patients diagnosed with this aggressive cancer.

## Data Availability

The original contributions presented in the study are included in the article/supplementary material. Further inquiries can be directed to the corresponding author.
